# An update on recent advances in targeted memory reactivation during sleep

**DOI:** 10.1038/s41539-024-00244-8

**Published:** 2024-04-15

**Authors:** Julia Carbone, Susanne Diekelmann

**Affiliations:** 1 Institute of Medical Psychology and Behavioral Neurobiology, University of Tübingen, 72076 Tübingen, Germany; 2 Graduate Training Centre of Neuroscience, International Max Planck Research School, 72076 Tübingen, Germany; 3 Department of Psychiatry and Psychotherapy, University Hospital Tübingen, 72070 Tübingen, Germany

**Keywords:** Sleep, Human behaviour, Consolidation

## Abstract

Targeted Memory Reactivation (TMR) is a noninvasive tool to manipulate memory consolidation during sleep. TMR builds on the brain’s natural processes of memory reactivation during sleep and aims to facilitate or bias these processes in a certain direction. The basis of this technique is the association of learning content with sensory cues, such as odors or sounds, that are presented during subsequent sleep to promote memory reactivation. Research on TMR has drastically increased over the last decade with rapid developments. The aim of the present review is to highlight the most recent advances of this research. We focus on effects of TMR on the strengthening of memories in the declarative, procedural and emotional memory domain as well as on ways in which TMR can be used to promote forgetting. We then discuss advanced technical approaches to determine the optimal timing of TMR within the ongoing oscillatory activity of the sleeping brain as well as the specificity of TMR for certain memory contents. We further highlight the specific effects of TMR during REM sleep and in influencing dream content. Finally, we discuss recent evidence for potential applications of TMR for mental health, educational purposes and in the home setting. In conclusion, the last years of research have provided substantial advances in TMR that can guide future endeavors in research and application.

## Introduction

Sleep is known to support the consolidation, i.e. the strengthening and integration, of newly acquired memories^1^. Memory consolidation during sleep is assumed to rely on the covert reactivation of newly encoded memory traces. This process involves the reprocessing and redistribution of the newly acquired information, together with its integration in the network of pre-existing memories. The hippocampus and neocortex are two brain structures that work in tandem for memory reactivation to occur^2,3^. New memories are assumed to be initially bound by the hippocampus for temporary storage. During subsequent sleep, memory traces are reactivated and redistributed for more permanent storage in the neocortex. During a night of sleep, the human brain undergoes different sleep stages that alternate in a cyclic manner. Human sleep is composed of rapid eye movement (REM) sleep and non-REM (NREM) sleep, which includes light sleep (stages N1 and N2) and deep sleep, also known as slow wave sleep (SWS, stage N3). Unique electrophysiological patterns characterize NREM sleep, most prominently neocortical slow oscillations (SO, 0.5–1 Hz), thalamocortical spindles (9–15 Hz), and hippocampal ripples (80–200 Hz). It is assumed that the precise temporal coordination between these oscillations form the basis for memory reactivation^4,5^.

**Fig. 1:**
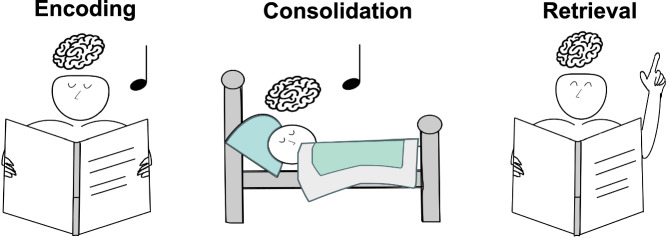
Targeted memory reactivation. In Targeted Memory Reactivation (TMR), learning content is associated with sensory cues, such as sounds, during encoding. During subsequent sleep, the same cues (e.g., sounds) are presented again to facilitate or bias memory consolidation in a certain direction. The effects of TMR on memory can be observed during retrieval (without presentation of the cue).

While memory reactivation occurs spontaneously in the sleeping brain, it can also be noninvasively reinforced with Targeted Memory Reactivation (TMR)^6,7^. TMR is a well-established technique to selectively stimulate specific memories to be reactivated during sleep using sensory cues linked to prior learning (Fig. 1). In TMR studies, specific cues like odors or sounds are presented during learning to become associated with the learning material. The same cues, or a subset of these cues, are then re-presented during subsequent sleep. Thereby, TMR can be used to directly manipulate memory reactivation and consolidation during sleep.

Our goal with this review is to present a brief and accesible overview of the most recent advances on the research of TMR, thereby giving an outlook on the potential of this tool for the research on basic mechanisms of memory formation as well as for applications in various areas. This review article builds on a previously published book chapter^8^ and includes the most recent TMR studies from the last 3-4 years following the publication of this chapter. Readers looking for other more systematic reviews and meta-analyses on TMR are referred to Lewis and Bendor^6^ and Hu et al.^7^.

In the following, we will first discuss recent advances in the use of TMR for different types of memories, such as declarative, procedural and emotional memories. Original TMR studies usually focused on the effect of TMR on strenghening these types of memories^9–11^. More recent studies explored other aspects of memory via TMR, such as the persistence of TMR effects over longer time periods^12,13^, the role of interference between cues^14^, and the possibility of inducing memory forgetting^15,16^. What is more, some studies combined TMR with other methodologial approches such as virtual reality settings^17,18^, functional magnetic resonamce imaging (fMRI) and intracranial recordings to investigate specific brain areas activated during TMR^12,13,19,20^. Additionally, recent studies combined TMR with closed-loop stimulation to test the best time point for cue delivery in the ongoing oscillatory activity^21,22^. While most studies in the field applied TMR during NREM sleep, we will also discuss recent research specifically looking into the effects of TMR during REM sleep and its influence on dream content^23^. Finally, we will discuss different applications of TMR for mental health treatments^24,25^, as well as in educational^26^ and home settings^27,28^.

In order to improve readability of this review, we have classified the recent studies into different sections. Importantly, some studies are described in more than one section. Table 1 provides an overview of all studies included in this review with the respective sections they are mentioned in.

**Table 1:** Recent TMR studies included in this review

	Sections	Reference
Schechtman et al.*, Commun. Biol*. (2021)	Declarative memories, Specificity of TMR	^14^
Whitmore et al., *J. Sleep Res*. (2022)	Declarative memories, TMR for forgetting	^28^
Schechtman et al.*, Sci. Rep*. (2021)	Declarative memories, TMR for forgetting, Optimal timing for TMR	^16^
Ngo & Staresina, *PNAS* (2022)	Declarative memories, TMR for forgetting	^22^
Siefert et al.*, bioRxiv* (2023)	Declarative memories, Specificity of TMR	^32^
Bar et al.*, Curr. Biol*. (2020)	Declarative memories, Specificity of TMR, Education	^33^
Gao et al., *Neurobiol Learn Mem*. (2020)	Declarative memories, Specificity of TMR	^34^
Neumann et al., *Sci. Rep*. (2020)	Declarative memories, Education	^26^
Vidal et al.*, Sci. Rep*. (2022)	Declarative memories, Education	^35^
Rakowska et al.*, Neuroimage* (2021)	Procedural memories, Optimal timing for TMR	^12^
Rakowska et al.*, bioRxiv* (2022)	Procedural memories, Optimal timing for TMR	^13^
Picard-Deland et al., *Learn. Mem*. (2021)	Procedural memories, TMR during NREM and REM sleep, TMR influencing dream content, Home applications	^37^
Nicolas et al.*, J. Sleep Res*. (2023)	Procedural memories	^39^
Veldman et al.*, Cereb. Cortex* (2023)	Procedural memories	^38^
Pereira et al.*, Neuroimage* (2022)	Emotional memories	^19^
Legendre et al.*, bioRxiv* (2022)	Emotional memories	^20^
Xia et al.*, Curr. Biol*. (2023)	Emotional memories	^41^
Hutchison et al.*, Commun. Biol*. (2021)	Emotional memories, TMR during NREM and REM sleep	^42^
Yuksel et al.*, bioRxiv* (2023)	Emotional memories, TMR during NREM and REM sleep	^44^
Schechtman et al.*, Sci. Rep*. (2020)	TMR for forgetting	^15^
Whitmore et al.*, npj Sci. Learn*. (2022)	TMR for forgetting	^46^
Whitmore et al.*, Learn. Mem*. (2023)	TMR for forgetting	^47^
Göldi et al.*, Sci Rep* (2019)	Optimal timing for TMR	^21^
Wang et al.*, J. Sleep Res*. (2022)	Optimal timing for TMR	^49^
Shimizu et al.*, Front. Hum. Neurosci*. (2018)	Optimal timing for TMR	^18^
Abdellahi et al.*, Neuroimage* (2023)	Optimal timing for TMR	^50^
Antony et al.*, Curr. Biol*. (2018)	Optimal timing for TMR	^51^
Cairney et al.*, Curr. Biol*. (2018)	Optimal timing for TMR	^52^
Schechtman et al.*, Cell Rep*. (2023)	Specificity of TMR	^54^
Forcato et al.*, Commun. Biol*. (2020)	Specificity of TMR	^55^
Carbone et al., *Learn. Mem*. (2021)	Specificity of TMR	^56^
Pereira et al.*, J. Neurosci*. (2023)	TMR during NREM and REM sleep	^58^
Abdellahi et al.*, Neuroimage* (2022)	TMR during NREM and REM sleep	^59^
Picard-Deland et al.*, J. Sleep Res*. (2022)	TMR during NREM and REM sleep	^60^
Borghese et al.*, Front. Psychiatry* (2022)	TMR during NREM and REM sleep, Mental health,Home applications	^17^
Abdellahi et al.*, Elife* (2023)	TMR during NREM and REM sleep	^61^
Wick & Rasch, *Learn. Mem*. (2023)	TMR during NREM and REM sleep	^36^
Carbone et al., b*ioRxiv* (2023)	TMR during NREM and REM sleep	^57^
Salvesen et al.*, PsyArXiv (2023)*	TMR influencing dream content	^62^
Haar Horowitz et al.*, Cogn*. (2020)	TMR influencing dream content, Home applications	^63^
Schwartz et al.*, Curr. Biol*. (2022)	TMR influencing dream content, Home applications	^23^
Talamini et al.*, Curr. Opin. Behav. Sci*. (2020)	Mental health	^24^
van der Heijden et al.*, Neurosci. Biobehav. Rev*. (2022)	Mental health	^25^
Weinhold et al.*, J. Sleep Res*. (2022)	Mental health	^64^
Gvozdanovic et al.*, Hum. Brain Mapp*. (2023)	Mental health	^65^
Chen et al.*, Curr. Psychol*. (2021)	Mental health	^66^
Ziqing et al.*, bioRxiv* (2024)	Mental health	^67^
Göldi et al.*, npj Sci. Learn*. (2019)	Home applications	^27^
Amores et al.*, Front. Psychol*. (2022)	Home applications	^68^

## Types of memory

### Declarative memory

Declarative memory refers to memories for facts and events that can be explicitly retrieved^29^. The effectiveness of TMR has been investigated across various paradigms for declarative memory, demonstrating its versatile application and potential as a memory-enhancing technique. Numerous studies have explored tasks involving word pairs, spatial navigation, associative learning, and other types of memory tasks^9,10,30^. In many TMR studies, this learning material is paired with distinct sounds or tones (e.g., the picture of a cat with the sound *meow*), with the sounds or tones then being presented again during subsequent sleep. In this way, single learning items can be paired with specific sounds, allowing for a more targeted reactivation of single items during sleep. Other studies include a learning session in the presence of an odor (e.g., the scent of roses)^31^. When this odor is presented again during subsequent sleep, it serves as a more general context cue that reactivates the entire learning session.

A widely used memory task for TMR is the object-location task, where subjects have to learn the location of an image coupled with a specific sound. Schechtman and colleagues^14^ showed that in this paradigm the beneficial effect of TMR is independent of the number of images that are associated with a specific sound. Pairing a sound associated with either just one image, two images or six different images resulted in comparable memory benefits for cued versus un-cued items. Another recent study found that memory for an object-location task can also be effectively enhanced with a device to perform TMR at home^28^. Other than the object-location task, TMR was found to enhance memory for pairs of semantically related words (e.g., DIET–CREAM)^16^, when the cue words were re-presented during sleep (e.g., DIET), as well as for associations between verbs and images^22^. Another study designed “satellite” objects containing similar and different features, showing that TMR improved memory for features unique to each individual satellite^32^. TMR was also effective in enhancing spatial memory, where participants had to associate words to either the right or left visual field of the screen^33^. More applied studies proved TMR to be successful, for example, in learning of a microeconomics class while listening to classical music^34^, as well as learning English vocabulary^26^ or a history lesson^35^ in the presence of a specific odor. Thus, TMR enhances memory performance after one night of sleep in various types of declarative memory, although some recent studies did not replicate this effect^36^.

### Procedural memories

Procedural memories consist of the development of perceptual and motor skills through extensive practice. Previous studies have tested the effects of TMR on this kind of memory after a night of sleep with mixed results^7^. More recent studies have looked at the effects of TMR on the persistence of procedural memories for the long-term. Rakowska and colleagues^12,13^ used a motor memory task that consisted of a bimanual (involving right and left hands) serial reaction time task (SRTT) with 12-item sequences being paired with high or low pitched tones. One of the sequences was reactivated during subsequent NREM sleep, while the other was not. Interestingly, while no effect of TMR was observed after 24 h, subjects showed better performance for the cued compared to the uncued sequence after 10 days, with this effect vanishing after another 6–8 weeks^12^. In a follow-up study, longitudinal structural and functional MRI showed that activity in the dorsal precuneus was related to the short-term effects of TMR (over 24 h), whereas sensorimotor regions support the long-term effects over 20 days^13^. In both studies, spindle density was further found to be higher during the cued compared to the un-cued sleep period and higher over the left versus right motor areas for the cue period. These findings suggest that TMR for procedural memory triggers a process that unfolds over the course of several weeks, possibly related to spindle activity.

Another approach uses virtual reality to explore procedural memory reactivation during sleep. Picard and colleagues^37^ applied a virtual environment in which participants were required to fly and accumulate points by traversing and avoiding floating items. A pleasant melody of four tones was played during the game and used afterwards for either TMR during a morning nap or a resting period. Performance overall improved when TMR was applied during REM sleep but not during SWS. Yet, spindle density was found to be associated with higher performance improvements when TMR was administered during SWS.

Another interesting approach uses somatosensory TMR by stimulating the effectors that were involved in a motor task, such as the fingertips^38^. Participants learned two sequences of a serial reaction time task (SRTT) in the MRI scanner, and later had a stimulation session during quiet rest where their fingertips received electrical stimulation alternating between the sequence and random stimulation. After 24 h including a night of sleep at home, participants showed faster responses for the reactivated compared to the non-reactivated sequence. MRI data revealed that regions recruited during initial motor learning were reactivated during the TMR session and hippocampo-cortical regions were modulated by the reactivation process. However, it remains to be elucidated whether somatosensory TMR during sleep is equally effective for motor learning.

A recent study in older adults observed no significant effect of TMR in improving motor memories^39^. Electrophysiological analyses suggested that responses to cueing are preserved in the ageing brain, however, the ability to differentiate between relevant and irrelevant stimuli seems to be impaired. Thus, the effects of TMR for procedural memories may depend on certain characteristics of the type of task, the time of testing, the presence of certain sleep parameters as well as the brain’s ability to detect relevant stimuli.

### Emotional memories

The effects of TMR for emotional memories and their neural correlates are still not well investigated. Non-invasive electroencephalography (EEG) has limited access to deep brain regions implicated in emotional memories such as the amygdala and orbitofrontal cortex (OFC). Two recent studies combined TMR with MRI and intracranial recordings, respectively, to better understand emotional memory reactivation during sleep.

Pereira and colleagues trained participants on an arousal rating task, where they were asked to rate picture-sound pairs from neutral to more negative^19^. The same picture-sound pairs were then applied in a modified version of an object-location task. In that task, it was previously found that SWS duration and spindles predict faster memory judgments for negative, but not neutral, picture locations after TMR^40^. Pereira and colleagues obtained fMRI recordings during testing after a night of sleep with TMR^19^, showing that TMR influences changes in brain activity in the amygdala and OFC. In particular, TMR modulated OFC activity in a valence-specific manner: cueing neutral items increased OFC activity, while cueing negative items decreased it. Moreover, the effect of cueing on amygdala activation was greater when more time was spent in REM sleep. Regarding TMR, surprisingly there was no cueing effect, neither for the negative nor neutral stimuli.

In a study by Legendre and colleagues, intracranial recordings were obtained from epileptic patients in brain regions implicated in emotional memories^20^. Patients were shown emotionally relevant (i.e., humorous) vs. emotionally neutral pictures, paired with different tones. When the tones were presented again during a subsequent nap, participants showed better memory for humorous pictures than neutral ones. Intracranial recordings revealed that the tones associated to humorous pictures enhanced SO and spindle activity in the OFC, paralleled with an increase in theta connectivity between the hippocampus and the OFC. Additionally, humorous pictures induced a similar time course of theta power than neutral pictures in all conditions except for the sleep group at testing, suggesting that TMR during the nap changed the memory traces of emotional pictures.

In a different line of research, TMR was applied for updating emotional memories, in attempt to test whether this technique can be used to modify unwanted memories, such as traumatic experiences^41^. Subjects learned associations between spoken pseudowords (e.g., GuXu) and negative images (e.g., a monster). During subsequent sleep, the aversive pseudowords were presented again together with either positive cues (e.g., cheering) or neutral cues. In the next morning, cues were rated as less aversive when they were paired with positive words during sleep, indicating that TMR changed the affective tone of unpleasant memories and made them less unpleasant. EEG analyses showed that theta power predicted affective updating, with larger theta power for positive words during cueing being associated with greater affective updating.

In an attempt to reduce emotional responses during sleep, Hutchison and colleagues^42^ performed TMR during REM sleep and SWS. Based on the sleep to forget, sleep to remember (SFSR) hypothesis, proposed by Walker and collaborators^43^, it was assumed that the dissipation of emotional charge relies on memory reactivation during REM sleep. Participants rated emotionally negative and neutral pairs of pictures and sounds for arousal both before and after a night of sleep, with half of the negative and half of the neutral sounds being played again either during REM sleep or SWS. As expected, TMR during REM sleep but not during SWS resulted in a stronger decrease of arousal ratings for negative pictures, which was driven by the largest decreases for the most negative stimuli. In another recent study on emotional memory, Yuksel and colleagues had participants learn the location of emotional pictures on a grid associated with specific sounds used for subsequent cueing during sleep^44^. Unexpectedly, reactivation of emotional stimuli was enhanced when cueing occurred during SWS but impaired during REM sleep.

Thus, recent studies provide behavioral and physiological evidence for differential effects of TMR on emotional memories. Depending on the procedure, TMR may either strengthen emotional memories or reduce the affective tone.

### TMR for forgetting

Traditionally, TMR has been used to strengthen memories and enhance memory performance. However, recent studies have begun to explore the intriguing possibility of using TMR to selectively weaken memories during sleep. This line of research is particularly relevant for clinical applications and the treatment of disorders associated with traumatic memories. The findings on emotional memory updating and the reduction of affective tone by TMR, as reported above, suggest that TMR could indeed be used to facilitate targeted forgetting.

Simon and collaborators introduced the idea of using TMR to promote forgetting in a study, in which participants were first trained to associate a tone to the act of forgetting, and subsequently learned object-sound-location pairings^45^. During SWS, object-sounds were presented paired to the forget-tone. One week later, participants recalled significantly fewer objects that were paired with the forget tone. More recent studies by Schechtman and colleagues continued exploring the possibility of inducing forgetting with TMR. In one study, they designed a task in which participants were instructed to memorize the location of some images while the locations of other images should be forgotten intentionally^15^. Whether or not a specific image should be remembered or forgotten was signaled by different sound cues. During a subsequent afternoon nap, sound cues used to signal forgetting were presented again. After sleep, memory performance was reduced for the to-be-forgotten images associated with the presented cues relative to those cues not presented during sleep. In a subsequent study, participants learned pairs of semantically related words (e.g., DIET–CREAM) and were then exposed to cue words (e.g., DIET)^16^. In a Think-no-think paradigm, participants were instructed to either recall (“think”) or suppress (“no‑think”) the associated word. The findings confirmed that suppression impaired retention of the corresponding target word (e.g., CREAM) when tested immediately after a 90‑min nap. However, TMR with one of two sounds conveying suppression instructions during sleep did not enhance suppression‑induced forgetting.

Interestingly, another series of studies explored the effect of TMR on memory when TMR cues disrupted sleep, suggesting that this procedure can lead to selective forgetting^28,46,47^. In one of these studies, Whitmore and colleagues trained subjects on object-sound associations and subsequently manipulated the intensity of the sound cues during sleep. They observed that reactivation with sound cues that induced sleep arousals selectively weakened the associated memories^47^. Thus, the possibility of forgetting with TMR should be investigated further in future studies.

### Optimal timing for TMR

Considering mixed findings on the effectiveness of TMR, a more recent approach suggests that TMR effects depend on the time point of cue application in relation to the ongoing brain oscillatory activity. This kind of manipulation is usually referred to as closed-loop or real-time TMR. Closed-loop stimulation during sleep was originally developed to enhance endogenous sleep rhythms (i.e., SO) by presenting sound clicks at a specific phase of the SO. The original study by Ngo and colleagues found that sound presentation in the up-state (or down-to-up transition) of the SO enhances the ongoing SO amplitude, increases associated spindle activity, induces another subsequent SO and improves sleep-dependent memory consolidation^48^. These findings suggest that the SO up-state might represent a particularly sensitive time window for the occurrence and induction of memory reactivation.

By combining closed-loop stimulation with TMR, recent studies explored the effects of precise cue presentation in the ongoing SO. Göldi and colleagues examined this question in a study where participants learned a Dutch-German vocabulary task^21^. They were then presented again with a selection of the Dutch words during subsequent NREM sleep, with the words being either presented during SO up-states, down-states or were not cued at all. Interestingly, only memory cues presented in the SO up-states improved subsequent recall performance, whereas down-state cueing did not result in a clear benefit when compared to the un-cued control condition. However, up-state cueing did not lead to superior performance when compared with down-state cueing directly. This finding is in line with another study by Wang and colleagues^49^, in which participants learned pairs of words, with the first syllable of each pair being subsequently presented either in the up-state or down-state of endogenous SO. No differences were found in memory performance between up-state and down-state cueing.

**Fig. 2:**
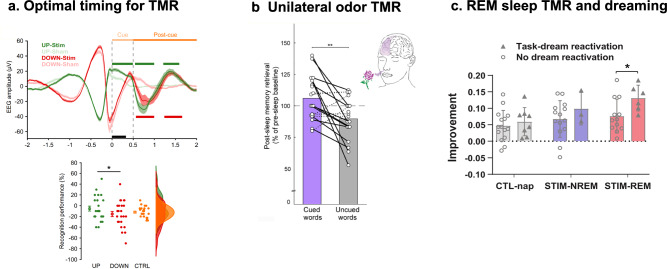
Important recent findings on TMR. **a** Optimal timing for TMR. Ngo & Staresina delivered sound cues at slow oscillation (SO) up-states (UP-stim) and down-states (DOWN-stim) compared to respective sham conditions. The upper panel shows the mean amplitude for SO in the UP-stim (green) and DOWN-stim condition, indicating the cueing (Cue, light orange) and post-cue periods (Post-cue, dark orange). Lower panel: Presenting the sound cues during SO up-states resulted in signficantly better memory performance for previously learned sound-word-image associations (UP, green) than when cues were presented in the SO down-state (DOWN, red). Reprinted from *Proc. Natl. Acad. Sci*. 119, Ngo & Staresina. Shaping overnight consolidation via slow-oscillation closed-loop targeted memory reactivation, e2123428119, Copyright (2022). **b** Unilateral odor TMR. Bar and colleagues designed a TMR approach to stimulate one brain hemisphere during sleep. Behavioral results for a declarative spatial memory task show that memory was selectively improved for words processed in the cued hemisphere (cued words, purple) compared to uncued words (gray). Reprinted from *Curr. Biol*. 30, Bar et al. Local Targeted Memory Reactivation in Human Sleep, 1435-1446.e5, Copyright (2020), with permission from Elsevier. **c** REM sleep TMR and dreaming. Picard-Deland and colleagues tested whether TMR and dream reactivations benefit whole-body procedural learning. Participants learned how to fly in a virtual reality setting in the presence of a melody, which was subsequently played again during a nap in either NREM sleep (STIM-NREM) or REM sleep (STIM_REM) compared to a control nap without TMR (CTL-nap). Performance improved particularly for those participants who received TMR during REM sleep and reported dreaming of the task (Task-dream reactivation). Reprinted from *Neurobiol. Learn. Mem*. 38, Picard-Deland et al. Whole-body procedural learning benefits from targeted memory reactivation in REM sleep and task-related dreaming, 107460, Copyright (2021), with permission from Elsevier.

Another recent study by Ngo and Staresina used a larger set of stimuli, including associations between 120 verbs and a set of 6 repeating images with associated sounds (e.g., car and engine starting)^22^. The 6 images and sounds were classified into 3 categories: objects, scenes and body parts. During NREM sleep, two sounds corresponding to two different categories were presented either during SO up-states or down-states, with a novel sound being used as control during both up- and down-states. Using this protocol, behavioral results revealed reduced overnight forgetting when cueing occurred during the SO up-state compared to the down-state (Fig. 2a).

EEG analyses from the previously mentioned studies revealed mixed findings. While Wang et al. observed no differences between up-state and down-state cueing^49^, Göldi et al. found that successful TMR during SO up-states was related to higher theta and spindle band activity than non-successful TMR, which was not evident for the down-state condition^21^. Ngo et al. observed that up-state cueing resulted in a prolonged SO up-state and stronger spindle power during the subsequent SO cycle following up-state cueing compared to down-state cueing^22^. Up-state cueing was even found to elicit reinstatement of target representations during the SO after sound offset, suggesting that up-state cueing induced reactivation of the associated memory representations.

Two other studies specifically targeted the up-to-down vs. down-to-up transition of SO for TMR application. Shimizu and colleagues applied TMR time-locked to the down-to-up-state transition of SO after participants navigated different routes in a virtual reality task in the presence of different sounds^18^. When sounds were presented in the down-to-up transition of ongoing SOs, average navigation time improved on the first day after learning. Moreover, fast spindle activity was increased in the down-to-up transitions compared with un-cued sleep. The second study by Abdellahi and colleagues directly compared the presentation of sounds associated with a procedural memory task (12-item SRTT as described above) during different phases of the ongoing SO^50^. Using a machine learning pipeline to predict the optimal time for TMR stimulation, they found that cues, which fall within the up-going transition of the SO, are more likely to elicit a classifiable reactivation.

Additionally, recent findings suggest that optimal timing for TMR also depends on the timing of cueing in relation to sleep spindles. Antony and collaborators showed in a first step that there is a refractory period between spindles of about 3–6 s^51^. Using real-time spindle tracking, the authors then presented TMR cues either within or right after this refractory period and found better memory improvement when cues were presented after the refractory period. Furthermore, a study by Cairney and colleagues showed that it is possible to decode the content of memory reactivation based on the TMR-evoked spindle activity, providing further evidence for the role of spindles in memory reactivation^52^. For a framework of the assumed memory function of spindle oscillations, the reader is referred to Antony and colleagues^53^. Together, these findings suggest that the timing of cue presentation in relation to the ongoing SO and spindle activity is critical for TMR effects, which should be considered in future TMR studies.

### Specificity of TMR

In research on TMR, it is still an open question to what extent cueing can specifically target single memories or certain aspects of a memory and whether cueing of one memory may interfere with the consolidation of other memories. Moreover, the underlying mechanisms behind TMR, such as the structure of the cue presented during TMR as well as the relationship of TMR with certain neuromodulators are still a matter of debate. A study by Bar and colleagues tested this question, taking advantage of the unique ipsilateral neuroanatomy of the olfactory system to perform TMR locally^33^. Participants learned a spatial memory task, i.e., associating words to either the right or left visual field of the screen, which elicited lateralized EEG markers of one brain hemisphere. During learning, bilateral stimulation of the olfactory system with an odor was delivered through a nasal mask covering both nostrils. When the odor was administered again specifically to one nostril during a subsequent nap, memory was selectively improved for words processed in the cued hemisphere (Fig. 2b). Time frequency analyses during TMR revealed spindle power and slow wave activity power increases during odor stimulation. Slow wave power (0.5–4 Hz) was lower in the cued hemisphere and correlated negatively with memory improvement for cued words. Interestingly, SO-spindle coupling changed locally such that spindles in the cued hemisphere peaked later and closer to the SO peak.

Another study tested whether multiple memories can be reactivated simultaneously or whether they would interfere with each other^14^. Participants learned an object location task, where they had to memorize the location of an image coupled with a specific sound. Images were classified to either small (1 item), medium (2 item) or large categories (6 items) (e.g., 6 different cat pictures in different locations were associated to the same *meow* sound). For those categories that were reactivated during subsequent sleep, memory performance was improved independent of the number of items per category. EEG analyses showed spindle and theta power increases upon reactivation, with the highest increase for sounds related to six items. Thus, it seems to be possible to reactivate multiple memories independently at the same time, suggesting that reactivation occurs in a simultaneous and promiscuous manner.

Another study tested the question whether memory reactivation drives memory transformation^32^. Siefert and colleagues applied an object category learning paradigm, where participants learned three categories of “satellite” objects before taking a 90 min nap. Each satellite had unique features as well as shared features with other members of its category. Moreover, each object had a specific name that was used for subsequent TMR. Interestingly, TMR improved memory for features unique to each individual satellite, while it impaired memory for features shared across satellites in the same category.

The question whether reactivation of some memories may also affect other related memories, was recently tested in a study on TMR and context reinstatement^54^. Participants were asked to invent idiosyncratic stories for different contextually bound sets, each set containing one image of a place and four objects. Subsequently, the objects were presented in different positions on a 2D grid associated to an object-related sound, while participants were asked questions related to the objects present in their story to keep the encoding context salient. Afterwards, participants had a nap and some of the sounds corresponding to a contextual set were presented via TMR. The results suggest that memory recall of non-cued objects was affected by contextually linked cued objects. The authors propose that individual memory reactivation during sleep may impact consolidation of memories sharing an associated encoding context.

Another study tested the effect of TMR when either complete or incomplete reminders are presented^55^. Participants learned the association between sounds and words, and where then presented with either the sound+word (i.e., complete reminders) or the sound+first syllable of the word (i.e., incomplete reminders). When participants slept for 40-min, both reminders were equally effective in stabilizing memories. However, when the sleep period was extended to 8 hours, only incomplete but not complete reminders stabilized memories. The authors propose that only incomplete reminders initiate long-term memory stabilization. In a subsequent pharmacological study, they modulated the GABAergic system via Zolpidem administration to evaluate changes in sleep oscillations and their functional relation to memory reactivation^56^. Using the same memory paradigm, they presented incomplete reminders and found that TMR with Zolpidem significantly enhanced memory performance when compared to placebo. Furthermore, Zolpidem increased the coupling of fast spindles and theta to slow oscillations, suggesting that GABAergic activity may be functionally implicated in memory reactivation processes during sleep.

### TMR during NREM and REM sleep

Most TMR studies have presented cues during NREM sleep, especially during SWS. However, it remains a matter of debate whether different NREM sleep stages may play different roles for TMR. Two recent studies directly targeted this question, comparing TMR during SWS and sleep stage N2^36,57^. Wick and Rasch implemented a vocabulary learning task that had already been used in previous studies from their group^27,30^. They observed no differences in recall performance between words reactivated during SWS and N2^36^. Furthermore, they failed to find an overall memory benefit of TMR over non-reactivated cues, questioning the robustness of TMR benefits. Similarly, Carbone and colleagues applied a sound-syllable-word memory paradigm that had been tested in previous studies^55,56^. Again, memory performance was not significantly different between cues presented during SWS and N2^57^. Interestingly, Wick and Rasch found that words played during N2 elicited stronger characteristic oscillatory responses when compared with SWS, whereas Carbone et al. observed higher evoked-response potential amplitudes for cues presented during SWS, and a higher density of SO and SO-spindle complexes during SWS compared to N2.

The effects of TMR during REM sleep have rarely been investigated. A few recent studies directly focused on TMR during REM sleep, when compared to NREM sleep stages. The study by Hutchison and colleagues (as mentioned in the section on emotional memory) compared the effects of TMR during REM sleep and SWS for the reduction of affective tone in an emotional memory paradigm^42^. Considering that REM sleep has been proposed to play a specific role in the consolidation of emotional memories by reducing the affective tone of these memories^43^, the authors hypothesized that TMR during REM sleep, but not SWS, would result in reduced arousal ratings for negative pictures. As expected, cueing the negative pictures during REM sleep reduced the arousal, while cueing during SWS did not. This effect was driven by the most negative stimuli.

Another study focused on the role of TMR for rule abstraction, in particular during REM sleep and SWS^58^. The experiment consisted of pairing abstraction problems with sounds to be used for TMR during the different sleep stages. The results revealed an improvement on abstraction problems for REM cues, but not for SWS cues, at the one week follow-up test. The authors propose that a sequence of plasticity events is initiated during REM sleep that requires more time to be completed. Focusing on emotional memory consolidation, Yuksel and colleagues compared TMR during SWS and REM sleep^44^. Contrary to the authors expectations, reactivation of emotional stimuli was enhanced during SWS but impaired during REM sleep.

Another study provided evidence for wake-like memory reactivation during REM sleep after TMR^59^. Machine learning classifiers were used to identify the reinstatement of wake-like memory patterns during REM sleep after participants were trained on a procedural SRTT task. TMR during REM sleep indeed elicited detectable reactivation, which was mediated by high theta activity and was partly temporally compressed and partly dilated compared to the wake experience. Although TMR during REM sleep did not directly improve SRTT performance, the amount of reactivation observed during REM sleep predicted overnight improvement on the SRTT.

Picard and colleagues published two subsequent studies, showing that TMR during REM sleep benefits procedural skill memory consolidation^37,60^. Participants completed a virtual reality (VR) flying task prior to and following a morning nap, during which tones related to the VR task were replayed in either SWS, REM sleep or wakefulness. The findings indicate that VR performance benefits most from TMR when applied during REM sleep.

Another applied TMR study delivered cues during REM sleep to treat social anxiety disorder^17^. The findings indicate an association between the number of auditory cues during REM sleep and a parasympathetic measurement of anxiety level (for more details see section on mental health). Thus, TMR during REM sleep may be effective for specific types of memory, mostly emotional memories and certain types of procedural memories. A recent study by Abdellahi and colleagues even provides evidence that TMR in REM sleep can elicit detectable reactivation, showing similarities with reactivation during NREM sleep stages^61^.

### TMR influencing dream content

A recent research area combines TMR methods with dream content studies to investigate the impact of external stimuli on dreams (for a systematic recent review on this topic refer to^62^). This line of research may eventually provide insights into the question whether and how memory reactivation during sleep is related to ongoing dreaming.

Two studies by Picard and colleagues (see above) combined TMR with a VR task in which participants were trained to fly in a virtual environment. The authors then explored procedural memory benefits and their relation to dream content^37,60^. REM sleep dream content related to the VR task (e.g., flying, accelerating) was found to be associated with better performance on the VR task (Fig. 2c). Moreover, TMR had a delayed effect on dream content that depends on the sleep stage. Participants dreamed more about the task 1–2 days later when TMR was applied in REM sleep and 5–6 days later when it was applied in SWS, compared to participants with no TMR.

A group of researchers has recently developed a wearable electronic device for dream incubation called ‘Dormio’^63^. This device makes use of the basic idea of TMR to present auditory information during a hypnagogic period, with the purpose of incorporating the auditory information into the dream content, a process called ‘targeted dream incubation’ (TDI). This tool can easily be applied in a home-setting but does not include EEG recordings. In a pilot experiment with this device, participants received instructions to think of a tree, whereas the control condition was asked to observe their thoughts. Subsequently, every time the device detected that the participant was falling asleep, it asked them to report verbally what was going through their mind and recorded their response. It was found that 67% of the awakenings in the experimental group incorporated the word ‘tree’.

Finally, a recent study applied TMR to influence the content of nightmares^23^. In an imagery rehearsal therapy (IRT) session, participants were instructed to consciously change the negative storyline of their current nightmares, which took place in the presence of a sound. Participants performed this IRT session in the evening at home and the sound was then re-presented during subsequent REM sleep. After two weeks of IRT with TMR participants reported less frequent nightmares and more positive dream emotions than the control group. The decrease of nightmares was sustained after three months, suggesting that TMR may also be applicable to treat certain mental disorders.

## Applications

In the last section we will introduce some of the most recent TMR applications. First, we will review a group of studies that explores TMR as a possible tool to treat mental disorders or to improve mental health. Next, we will describe recent studies using TMR for educational purposes. Finally, we present studies introducing the possibility of using TMR at home outside of laboratory settings.

### Mental health

Two recent review articles have discussed potential applications of TMR for traumatic memories^25^ and affective disorders^24^. The first review explored the potential benefits of TMR for the treatment of Post-Traumatic Stress Disorder (PTSD), while the second one proposes TMR as a sleep-based treatment for maladaptive emotional memories such as in phobias, addictions or PTSD. In the following, we will only highlight the most recent studies not included in the aforementioned reviews.

A clinical study included stable and medicated schizophrenia patients in a double-blind sham-controlled TMR design^64^. Memory performance was assessed by a verbal (word pairs) and non-verbal (complex figure) memory task. TMR evoked an electrophysiological response similar to that in healthy participants, increasing SO and spindle coupling during stimulation. However, there was no memory performance improvement after sleep.

A study by Borghese and colleagues focused on TMR to treat social anxiety^17^. Participants with social anxiety had two exposure therapy sessions where they had to give a public talk in front of a jury in a virtual reality setting. At the end of the session, half of the participants (TMR group) received positive feedback paired with a sound, which was subsequently presented again for one week during sleep at home. Although TMR did not affect subjective measures of anxiety when compared to a control group, the number of auditory cues during REM sleep as well as REM sleep duration was associated with a parasympathetic measurement of anxiety level (i.e., root mean square of successive differences between normal heartbeats), suggesting that more TMR cues and longer duration of REM sleep reduced the physiological anxiety reactions.

Another study examined the effects of sleep and TMR on traumatic memory development^65^. Participants were presented with an odor while watching a trauma film. The same odor was then presented again during subsequent SWS to facilitate memory integration. The findings show that sleep per se reduced the number of intrusive traumatic memories compared to wakefulness, however, there was no additional benefit of TMR.

Another line of research investigated TMR in relation to positive self-images, considering that the self-concept can affect mental health in various ways. Chen and colleagues combined a self-esteem training with audio cues, which were then re-presented during a subsequent 90-min nap^66^. Results showed that TMR increased individuals’ implicit self-esteem level, which was still maintained one week later. Ziqing and colleagues tested whether TMR can enhance positive self-evaluative memories^67^. Participants first had to rate positive and negative personality traits in relation to themselves. They then performed a Go/NoGo task, in which they were presented with positive personality traits (Go traits, e.g., brave, kind) that required them to press a button as quickly as possible. When half of the positive traits were presented again during NREM sleep in a subsequent nap, the recall of positive traits that were strongly memorized before sleep was increased, suggesting that pre-TMR self-evaluative memory strength modulated TMR benefits. The strongest memories thereby elicited significantly larger sigma power changes. Thus, TMR may be applicable in changing memories that are directly or indirectly linked with mental health.

### Education

In the last few years, several studies tested the applicability of TMR in a school setting to improve learning in students. Neumann and colleagues applied TMR with an odor in the class room of a 6th grade^26^. Students were instructed to study German-English vocabulary at home at their desk in the presence of an incense stick and to put the stick next to their bed at night while sleeping. This procedure resulted in superior performance in a subsequent vocabulary test when compared to students who did not receive the odor TMR. Effect sizes in the TMR groups were quite large (d = 0.6–1.2), indicating that TMR does not only work in a lab environment but also in a regular school setting. The authors also showed that TMR works effectively when presented continuously during the entire night rather than only during certain sleep stages.

Using a similar study design, Vidal and colleagues applied TMR in secondary school students who had a history lesson in the presence of an odor dispenser^35^. When students were presented with the same odor during the night at home, compared to a different odor in the control condition, memory performance of the history class was significantly improved. In another study, undergraduate students had a microeconomics class while listening to classical music^34^. During subsequent SWS, students were re-presented with either the classical music (TMR) or a control noise, showing that TMR increased the probability of passing the test with a grade of 70 or more. Interestingly, this benefit was only seen in female students but not in males, and the benefit was lost at a 9-month follow-up test. EEG analyses found that frontal theta activity was higher during SWS for the TMR group when compared to the control condition and greater theta activity was associated with better subsequent test performance as well as with less forgetting after 9 months in females. These findings provide convincing evidence for beneficial effects of TMR in educational settings.

### Home applications

In the previous paragraphs, we have already mentioned a few studies that successfully applied TMR in a home setting. These studies were possible due to the recent development of devices for the application of TMR outside the lab. Borghese et al. applied TMR at home in patients with social anxiety using a wearable headband device that automatically identified sleep stages and administered the TMR sounds during REM sleep^17^. They made use of an already existing headband designed by the company Dreem (Dreem SAS, Paris, https://dreem.com/), where sounds can be delivered via bone-conduction transducers integrated in the headband. Schwartz and colleagues likewise used the Dreem headband to treat patients with nightmare disorder across a 2-week period at home^23^. Two more studies effectively applied TMR with odors at home using incense sticks^27^ and odor dispensers^35^ without any EEG monitoring to present learning-associated odors during sleep at home in school students.

More recent studies have tested potential boundary conditions for home-applications of TMR. Göldi and Rasch performed unsupervised TMR at home to improve foreign vocabulary learning^27^. During three consecutive nights, participants used an mp3-player to play previously learned foreign vocabulary during sleep, without any control of sleep stages or awakenings. Per night analyses revealed that memory benefits by TMR were significant only in the third night, indicating that sleep disturbances and habituation to the sounds might be critical factors for the success of unsupervised TMR in a home setting.

Several groups of researchers have developed different devices for applications of TMR outside the lab. One previously mentioned device, i.e., ‘Dormio’, was developed by Horowitz and colleagues. Dormio is a wearable device for dream incubation designed to be used at home^63^. It consists of a hand-worn sleep tracker and an associated app used to communicate with users and record dream reports.

Whitmore and collaborators have developed ‘Sleepstim’, a system to perform TMR at home^46^, including a smartwatch to collect movement and heart-rate data, plus a smartphone to emit auditory cues. A machine-learning model is used to identify periods of deep sleep to trigger TMR sounds. In two experiments using SleepStim, the spatial memory benefit of in-laboratory TMR studies was tested. Participants learned object-locations associated to a sound and half of the objects were reactivated during three subsequent nights. The results showed that the memory benefit of TMR was dependent on sound intensity and sleep stage, with greater improvements when sound intensity was lower and when cues were delivered in SWS.

Another research group has developed a device for odor TMR at home^68^. ‘Ezzence’ is an olfactometer for home settings that can be controlled with the smartphone to release odors in real-time during specific sleep stages. Feasibility of the device was tested in a study where half of the participants slept at home in the presence of a lavender scent compared to a control condition without any interventions. Results showed that participants were satisfied with the device and found it easy to use. Moreover, participants reported better sleep as well as better mood in the next morning. Future studies will have to test the applicability of this device for TMR.

## Conclusions and limitations

The last 3-4 years of TMR research have provided substantial advances in understanding the effects, mechanisms and applicability of TMR as a tool to influence memory consolidation during sleep. Memory strengthening effects continue to be found across multiple memory systems, such as declarative and procedural memories. There is also increasing evidence for the potential of TMR to change emotional memories. More recent TMR studies raised other interesting questions, such as the role of different sleep stages, the importance of considering interference between cues, or even the possibility of using TMR for memory forgetting. Auditory cueing studies gained popularity by opening the opportunity to consider the best time point for stimulation in the ongoing oscillatory activity (i.e., closed-loop TMR), suggesting the up-state or down-to-up state transition of the SO as well as the periods outside of the spindle refractory period to be the most promising time windows for cue delivery. Meanwhile, odor cueing remains resourcefull as it can be easily transferred to more realistic scenarios. The last years have seen an upsurge of research on meaningful applications of TMR, confirming applicability in educational and clinical settings as well as at home.

Despite the fast advances in this field, there are still a number of open questions that need to be addressed in future research. One of the most interesting directions for future research will be the role and interaction of different sleep stages for memory reactivation. Particularly, the role of reactivation during REM sleep and its connection with emotional memories and dreaming should be further explored. Another open question relates to potential boundary conditions and critical factors determining whether TMR is effective or not, such as the timing of cue presentation or the number of items associated with single cues. Finally, the field would benefit from more research on potential applications of TMR in various settings to foster the transfer of promising basic research findings to much-needed real-life applications.

However, TMR research also faces important limitations. Several studies have recently failed to find a significant difference between reactivated and non-reactivated memories^39,69^. Future research should take greater efforts to replicate important findings in the field. Moreover, most published TMR studies include rather small sample sizes, challenging the possibility of comparing published work. Future TMR studies should include larger sample sizes and multi-lab studies of established paradigms, ideally with preregistration. Additionally, transferring laboratory experiments into real-life situations, such as educational settings, has some limitations. For example, unsupervised TMR sessions at home may lead to sleep arousals or awakenings (e.g., when sounds are too loud), which may hinder memory improvement or even weaken the associated memories. These limitations need to be considered when assessing TMR effects.

### Reporting summary

Further information on research design is available in the Nature Research Reporting Summary linked to this article.

### Supplementary information

Reporting summary
